# Fatal Effects from the Acetate of Lead Given in Large Amount for Phthisis

**Published:** 1840-10

**Authors:** 

**Affiliations:** Mulhouse


*Fatal effects from acetate of lead given in large amount for Phthisis Pulmonalis.* By Dr. Bicking, of Mulhouse.

Although we possess a large number of observations in which the acetate of lead has been given with success, or, at least, without serious accident, for phthisis pulmonalis, there are others in which troublesome results have followed from the prolonged use of this medicine, and, on this point, Dr. Bicking cites the following case:

Ferdinand R——, aged fifteen years, subject, during his younger days, to attacks of scrofula, suffered several times from affections of the chest, and finally became consumptive. Having arrived at an advanced stage of this affection of the lungs, accompanied with hectic fever, sweats, and colliquative diarrhœa, without any remedy having power to arrest or alleviate its course, Dr. Bicking commenced with the use of acetate of lead.

I gave, says he, to the patient, a quarter of a grain of acetate of lead reduced to powder, along with sugar of milk, four times a day, during a certain time. Under its influence I obtained a marked amendment in the course of the morbid symptoms; the fever, the sweats, the dejections, and cough diminished. The purulent expectoration likewise diminished without any increase of oppression; this advantageous result induced me to continue the treatment, and, during six weeks, I successively increased the dose of the medicine, so that at the end of this time the patient took two grains of the acetate of lead during the day.

At this period, the patient experienced a marked relief, troubled from time to time with some returns of the same general symptoms from which he had been relieved, and, at each return, I employed, with success, the same means. In twelve weeks every trace of phthisis had disappeared, and the child who returned to school, was no longer subject to medical treatment. He had taken, in the course of the disease, nearly an hundred and thirty grains of acetate of lead, without any poisonous or even hurtful effect.

In the meantime, he could not regain entirely, his health; his strength declined, he became thin and pale, and his pulse frequent; frequent difficulty of respiration, pains in the chest, and an obstinate cough of irritation. I was apprehensive of an immediate renewal of the consumptive attacks. My fears were realized, but in another manner.

One month after the appetite gradually failed, the hypochondrium became affected with painful spasms; the stools were rare and painful; the skin over the whole body became blueish; the conntenance became puffed up and hot, the hair fell out; soon a convulsive cough supervened, accompanied by great difficulty of respiration, and burning pains in the chest, to which succeeded partial paralysis of the feet. This state remained fourteen days. One evening he experienced a violent access of fever, with heaviness of the head, paralysis of the eyelids, and convulsions in the face and extremities.

All remedies were unavailable; the patient remained insensible, in stupor or delirium; he died the third day after, but no autopsy was made.—C. W. *Hufeland's Journ. des Practs. Heilk.* 1839.

The publication of this notice should not, however, lead to the belief that the medicinal use of acetate of lead is always followed by fatal effects. We believe that it is necessary to study the therapeutic action of this acetate; we know that large quantities of this salt have been taken, in several cases, and we have seen Dr. Bricheteau treat, with pills of acetate of lead, Mademoiselle C—— S——, who was considered to be affected with consumption. This treatment was followed by great success, and the lady is now married, and has several children, and enjoys a good state of health. A. C.

*Journ. de Chim. Med.*

The above case is one of those rare occurrences in which a remedy, in many cases of great advantage, has been followed by lamentable consequences from its prolonged use. It may be advantageous to enquire to what cause such effects may be owing. In addition to the observations in the note of M. A Chevallier, it may be asked, may not this effect arise from the formation of a carbonate of lead in the stomach of a patient whose digestive organs are much debilitated? Although pure neutral acetate of lead is not affected by free carbonic acid, nevertheless the acetate of the shops is always more or less affected by the presence of this gas, solutions always being cloudy when not made with distilled water. This is due to the loss of a portion of acetic acid, consequent on the exposure of the crystals to the air. This exposure is followed by an opacity of the external crystals by efflorescence, but that, in this instance, the result is not entirely owing to the loss of water of crystallization may be concluded from the strong smell of acetic acid which is exhaled by a jar of acetate of lead when recently unstopped. In this country, the acetate is commonly used in much larger doses than is here stated: a very common prescription, especially in dysentery, cholera morbus, &c., being two or three grains of acetate frequently repeated, and it is rare indeed to hear of any injurious effects therefrom. The custom, however here, is to unite, in the same prescription, a greater or lesser quantity of opium, as a means of obviating any injurious effects of the remedy, as well as to fulfil other indications in the above diseases. That it may have this tendency, will be obvious in considering the reactions which will be consequent on the mixture of these two ingredients. The meconic acid of the bimeconate of morphia would first neutralize any excess of oxide of lead in the acetate, and then the remainder would unite with more of the lead, and liberate acetic acid, thus forming an insoluble meconate of lead, which would pass through the intestines and be discharged. This remedy, however, is not employed to the same extent here in chronic cases, where a long continued use is required to produce its full effect, as it is in Germany. In this latter country it is very highly recommended in consumptive affections, and effects may result from its prolonged use, which would not appear from a greater amount of the remedy given in a much shorter time. R. B.
*American Journal of Pharmacy.*

